# A fiber-optic spectroscopic setup for isomerization quantum yield determination

**DOI:** 10.3762/bjoc.20.150

**Published:** 2024-07-22

**Authors:** Anouk Volker, Jorn D Steen, Stefano Crespi

**Affiliations:** 1 Department of Chemistry - Ångström Laboratory, Uppsala University, Box 523, 751 20 Uppsala, Sweden; 2 Faculty of Science and Engineering, University of Groningen, Nijenborgh 4, 9747 AG Groningen, The Netherlands

**Keywords:** isomerization, molecular photoswitches, photochemistry, photon flux, UV–vis spectroscopy

## Abstract

A spectroscopic setup for isomerization quantum yield determination is reported. The setup combines fiber-coupled LEDs, a commercially calibrated thermopile detector for measurement of the photon flux, and a fiber-coupled UV–vis spectrometer. By solving the rate equations numerically, isomerization quantum yields can be obtained from the UV–vis absorption spectra. We show that our results for the prototypical photoswitch azobenzene are in excellent agreement with the literature. The analysis of the errors showed that the quantum yields determined using this method are in the same order of magnitude as when using actinometry, thus demonstrating the reliability of our setup.

## Introduction

Photoswitches are molecules that can undergo a light-driven structural rearrangement to populate a metastable state of the initial reactant. This isomerization can be reversed by light of a different wavelength or thermal stimuli. Depending on the nature of the reactant, different classes of photoreactions can be utilized: double bond isomerizations, electrocyclization, cycloadditions, and electron-, hydrogen-, or group transfer [1].

Among the different switches known in the literature, azobenzene is a textbook example of a photoswitch operating via a double bond isomerization mechanism, with the first reports of its *trans*-to-*cis* interconversion under direct sunlight dating back to the work of Hartley in 1937 [2]. Over the decades, azobenzene and its derivatives [3–4] have found numerous applications in the development of novel materials [5–6], photopharmacology [7–9] but also as actinometers [10–11], due to the large geometry changes upon isomerization [12] and easily accessible derivatives [1,7,13–14].

**Figure 1:**
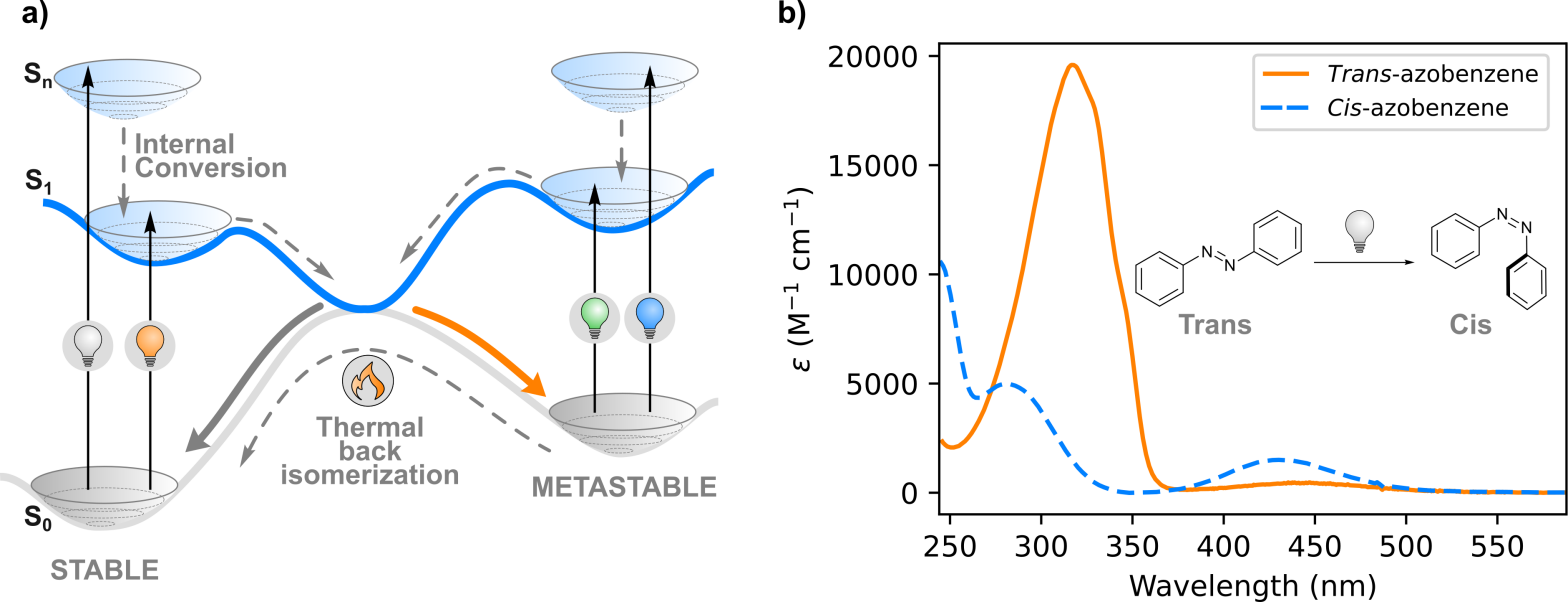
a) Schematic overview of a photochemical isomerization and b) absorption spectra of the isomers of azobenzene.

The absorption spectra of the two isomers each show distinct bands, as shown in Figure 1. For *trans*-azobenzene, there is a π→π* band around 320 nm and a very weak (symmetry-forbidden) n→π* band around 440 nm. *cis*-Azobenzene shows two bands at 240 and 280 nm, and the n→π* transition is slightly more allowed than for *trans*-azobenzene [15]. As the bands of *cis*- and *trans*-azobenzene overlap, irradiation results in a photostationary state (PSS) which is a mixture of the isomers. The distribution of the isomers at the PSS differs for irradiations at different wavelengths [16]. Irradiation at 340–350 nm, where the ratio ε*_trans_*/ε*_cis_* is the highest, results in the formation of >95% *cis*-azobenzene [17].

The quantum yields (Φ) of isomerization of this process have been determined using different irradiation wavelengths, solvents, and methods [18–24]. One of the most interesting properties of azobenzene is the dependence of the Φ on the irradiation wavelength [25]. Clear differences are reported between irradiation of the different absorption bands, due to the so-called anti-Kasha behavior of this molecule, i.e., the wavelength dependency of the quantum yield of reaction [22,24].

Despite its textbook example status, the photoisomerization of azobenzene is still under active investigation. Over the past two decades, both experimental [23,26] and theoretical work [27–32] has been performed to explain the differences in quantum yield. Not only the exact details of the photoisomerization are still under debate, but also the absorption spectrum of *cis*-azobenzene has been redetermined as recently as 2017 [17]. With the re-evaluated values for the molar absorptivities, the Φ of azobenzene were recalculated by the same group immediately afterwards [24].

As shown by Vetráková et al. [17], the spectrum of *trans*-azobenzene can be easily obtained by careful preparation of the sample in the dark. The absorption spectrum of *cis*-azobenzene, however, is challenging to obtain via direct measurement, due to its intrinsic thermal instability and sensitivity to ambient light. The authors showed that the *cis*-azobenzene previously isolated by extensive irradiation [2], liquid chromatography [22], or preparative TLC [21,33] contained some of the *trans*-isomer. Their work highlights the care that needs to be taken to obtain correct values for the molar absorptivities of *cis*-azobenzene. This conclusion holds for any photoswitch where only one isomer can be isolated successfully. During the measurement and at the PSS, the relative concentrations of the two isomers are unknown. The total absorbance (*Abs*_obs_) at a given total concentration (*c*) of the sample is a linear combination of the absorbance of each isomer, as illustrated by Equation 1.


[1] $$Abs_{\text{obs}}=c\cdot Abs_{trans}+(1-c)\cdot Abs_{cis}$$

The value for *c* at the PSS can be found using other spectroscopic methods, such as NMR spectroscopy [17] or the TEM (for Thulstrup, Eggers, and Michl) method [34–35]. With a known value for *c*, the spectrum of the second component can be found by a simple scaled subtraction [14]. Another powerful method to obtain the spectrum of the second isomer is global and target analysis, where time-resolved spectra can be analyzed to find the number of spectral components and the evolution of their concentration over time [36–37]. Additional information, such as a kinetic model describing the components and the non-negativity constraint on the absorbance, can help to guide the decomposition of the data into chemically meaningful concentration traces and species-associated spectra (SAS) [38]. Global and target analysis is one of the methods employed in recent literature to find the spectrum of *cis*-azobenzene [17].

To calculate the quantum yields, both the molar absorptivities of the isomers and the number of photons absorbed by the sample needs to be known very accurately. Utilizing chemical actinometers remains the most used method for the determination of photon flux, i.e., the number of photons impinging a unit area of the sample in a unit of time [10]. Potassium ferrioxalate [39–40] is one of the most used actinometers while also some azobenzene derivatives have been reported as reaction quantum yield standards [11,22]. Although new chemical actinometers are being proposed [41–42], actinometry is dependent on quantum yields determined decades ago [10,43]. Using chemical actinometers is often labor-intensive, and measurements are restricted to irradiation wavelengths where the actinometer absorbs. With high-quality and reliable thermopile and solar cell detectors now being commercially available at a reasonable cost, instructions for building laboratory apparatus for quantum yield determination using direct measurement of photon flux using solar cell detectors have been reported by Riedle and co-workers [44–45]. With the design reported by Stadler et al. [46], on the other hand, it is possible to follow the isomerization in operando, while the photon flux can be obtained by disassembling the setup and reading the power of the excitation source using an integrating sphere.

In this paper, we introduce a relatively simple setup that combines the positive features of the aforementioned designs, which can be used to determine the forward and backward quantum yields of molecular switches without the need for actinometry, by combining fiber-coupled LEDs with a commercially calibrated thermopile detector and following the isomerization of a molecular switch by UV–vis absorption spectroscopy in operando*.* We provide all the information necessary to build the setup as well as the scripts and examples to retrieve the quantum yields of forward and backward isomerization of a molecular photoswitch following the evolution of the absorption in the low absorption regime [47] upon irradiation.

## Results and Discussion

Azobenzene was chosen as the model compound for testing, as the recent work by Vetráková et al. [17] and Ladányi et al. [24] has provided us with very reliable molar absorptivities and quantum yields to use for validating our methods.

A molecular photoswitch generally has a stable state A and a metastable state B, which would be the *trans-* and *cis-*isomers, respectively, for azobenzene. The rate equations of the isomerization can be described in terms of the quantum yields and the molar absorption coefficients. It is possible to show that the change in the concentration of A is given by Equation 2 [47]:


[2] $$\frac{d[\text{A}]}{dt}=-\frac{\Phi_{\text{A}\to \text{B}}\cdot q_{\text{A}}}{N_{\text{A}}\cdot V}+\frac{\Phi_{\text{B}\to \text{A}}\cdot q_{\text{B}}}{N_{\text{A}}\cdot V}+k_{\text{B}\to \text{A}}[\text{B}]$$

The first term gives the photochemical reaction from A to B, which is governed by the number of photons absorbed at a given wavelength, *q*_A_, as well as the quantum yield Φ_A→B_ for this reaction. *N*_A_ is Avogadro’s number, *V* is the total volume of the sample. When the *cis*-isomer absorbs at the same wavelength, the reverse reaction will happen photochemically as well, as described by the second term. The last term describes the thermal reaction converting *cis-*azobenzene into the stable *trans*-azobenzene. For the selected example, the rate constant for the thermal back isomerization is 7.2∙10^−7^ s^−1^ at 20 °C [24]. This value is generally disregarded for routine experiments where the isomerization happens in the minute timescale, however we explicitly included it in our quantum yield determination for better accuracy.

Using the Beer–Lambert law, the light absorbed by the sample, *Abs*_tot_, in a setup with a given path length *l* can be expressed as the sum of the absorbances of the single species A and B and consequently in terms of the concentrations of A and B and their respective molar absorption coefficients (ε_A_ and ε_B_):


[3] $$Abs_{\text{tot}}=Abs_{\text{A}}+Abs_{\text{B}}=l\varepsilon_{\text{A}}[\text{A}]+l\varepsilon_{\text{B}}[\text{B}]$$

For absorbances below 2, not all photons are absorbed when passing through the sample. In this case, the number of photons absorbed by a species (*q*_A_ for species A in the following example) can be given by Equation 4 [47]:


[4]

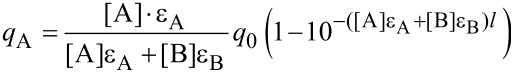



Where *q*_0_ is the total number of photons absorbed by the sample. Combining Equations 2 and 4, the rate equations for the system are given by Equation 5 and Equation 6:


[5] $$\begin{array}{l} \frac{d[\text{A}]}{dt}=\frac{1}{V}\frac{q_{0}}{N_{\text{A}}}\frac{1-10^{-\left([\text{A}]\varepsilon_{\text{A}}+[\text{B}]\varepsilon_{\text{B}}\right)l}}{[\text{A}]\varepsilon_{\text{A}}+[\text{B}]\varepsilon_{\text{B}}}\\ \left(\Phi_{\text{B}\to \text{A}}[\text{B}]\varepsilon_{\text{B}}-\Phi_{\text{A}\to \text{B}}[\text{A}]\varepsilon_{\text{A}}\right)+k_{\text{B}\to \text{A}}[\text{B}] \end{array}$$

[6] $$\frac{d[\text{B}]}{dt}=-\frac{d[\text{A}]}{dt}$$

So far, the spectral shape of the excitation light source (an LED in our case, vide infra) has not been explicitly considered and has been assumed to be monochromatic. Although this approximation is effective for many light sources with narrow bandwidths, the emission spectrum can be included in the rate equations. This correction is done by multiplying Equation 5 by the spectral distribution *f*(λ) of the light source and integrating over all wavelengths [45]:


[7] $$\begin{array}{c} \frac{d[\text{A}]}{dt}=\\ -\;\Phi_{\text{A}\to \text{B}}\int \frac{[\text{A}]\varepsilon_{\text{A}}}{[\text{A}]\varepsilon_{\text{A}}+[\text{B}]\varepsilon_{\text{B}}}\frac{q_{0}\lambda f(\lambda )}{VN_{\text{A}}}P\;d\lambda \\ +\;\Phi_{\text{B}\to \text{A}}\int \frac{[\text{B}]\varepsilon_{\text{B}}}{[\text{A}]\varepsilon_{\text{A}}+[\text{B}]\varepsilon_{\text{B}}}\frac{q_{0}\lambda f(\lambda )}{VN_{\text{A}}}P\;d\lambda \end{array}$$

where


[8] $$P=1-10^{-\left(\left[\text{A}]\varepsilon_{\text{A}}(\lambda )\;+\;[\text{B}]\varepsilon_{\text{B}}(\lambda \right)\right)l}$$

### Setup

**Figure 2:**
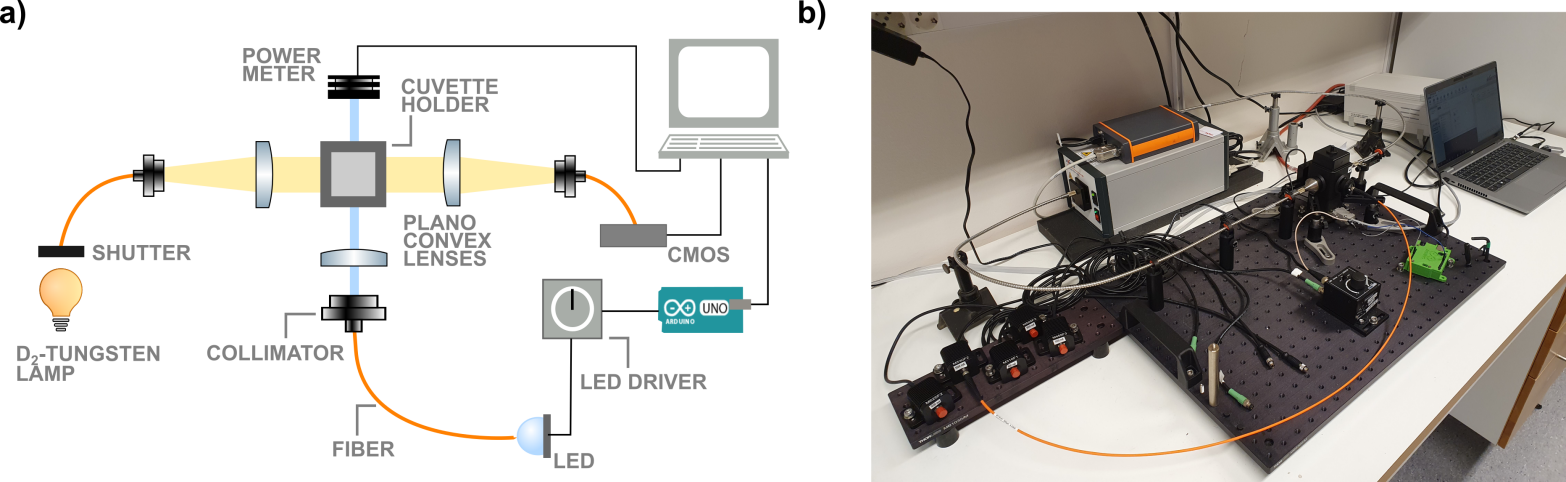
a) Scheme of the setup and b) picture of the setup.

The UV–vis irradiation experiments were performed using a setup assembled in-house, see Figure 2 and Supporting Information File 1. A Quantum Northwest Luma 40 Peltier-based temperature-controlled cuvette holder with an insulator jacket with four optical windows was used to allow irradiation perpendicular to the spectrometer light path. The light source is an Avantes AvaLight-DH-S-BAL light source coupled with a 400 µm fiber (Avantes FC-UVIR400-1-BX) to an SMA-to-SM1 fiber adapter (Thorlabs CVH100-COL) containing a plano-convex lens (20.1 mm focal length, Thorlabs LA4647) in a 1 inch diameter lens mount (Thorlabs LMR1S/M). The transmitted light is collected by a plano-convex lens (20.1 mm focal length, Thorlabs LA4647) in a 1 inch diameter lens mount (Thorlabs LMR1S/M) contained in an SMA-to-SM1 fiber adapter (Thorlabs CVH100-COL) connected to a 400 µm fiber (Avantes FC-UVIR400-1-BX). The spectrometer we used is an Avantes AvaSpec-ULS2048CL-EVO-RS. The light path for the irradiation consists of a fiber-coupled LED (Thorlabs M340F4, M395FP1, M455F3) coupled to a 600 µm fiber (Thorlabs M114L01) to an adjustable fiber collimator (Thorlabs CFCS5-A) mounted on an adapter (Thorlabs AD15F2) in a 1 inch diameter lens mount (Thorlabs LMR1S/M). On the opposite side of the cuvette holder, a power meter (Thorlabs PM16-401) is mounted. The LED is powered by an LED driver with trigger mode (Thorlabs LEDD1B T-cube) controlled using an Arduino UNO R3 connected to a PC.

The AvaLight-DH-S-BAL light source is connected to the spectrometer via an interface cable (Avantes IC-DB26-RM), which allows the proprietary software AvaSoft from Avantes to control the internal shutter of the light source via a PC. With this configuration, starting a single measurement in AvaSoft sends a TTL signal to the light source that opens the shutter, which is followed by the acquisition of a spectrum, and finally a TTL signal is sent to close the shutter again. By utilizing scripts we developed (see Supporting Information File 1), it is possible to precisely control the timing of irradiation of the sample with the LED and the perpendicular illumination with the UV–vis probe. In this way, when a UV–vis spectrum is recorded, the shutter of the D_2_-tungsten lamp is open and the excitation LED is switched off for the entirety of the measurement (average of 500 spectra acquired for 1.0 ms). When the measurement is finished, the script closes the shutter and the LED is switched on for a given amount of time. This sequence can be easily looped to obtain a desired illumination sequence (see Supporting Information File 1 for more detailed information on the loops and exact timings). The shutter prevents undesired illumination from the UV–vis probe that could influence the measurement altering the kinetics of isomerization, a known problem for similar setups [24].

**Figure 3:**
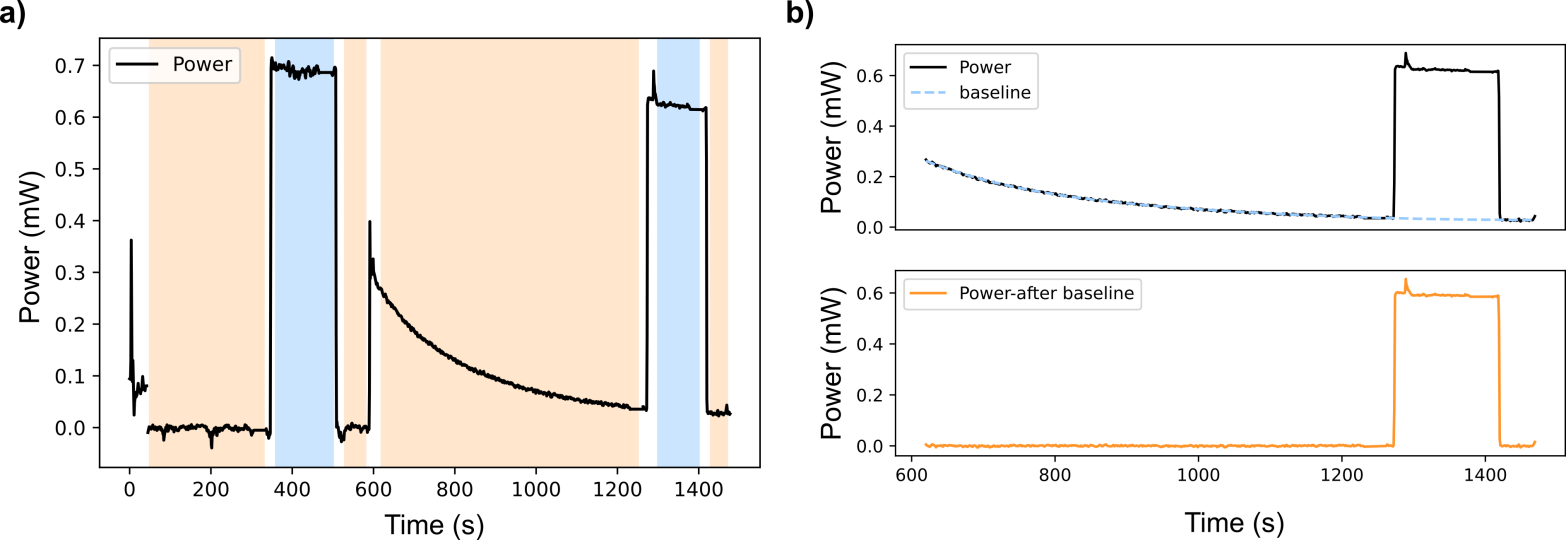
A visual example of the power determination. a) Power without any elements (left) and with insulated jacket and cuvette (right side, the change in baseline is noticeable), b) power with insulated jacket and cuvette after baseline subtraction.

The power readings from the thermal power sensor were recorded by using Thorlabs’ Optical Power Monitor (OPM) software. The resulting data showed some fluctuations depending on environment temperature and air movement. The background power was therefore monitored to get an accurate power reading before turning on the LED. The raw data was then fitted with a simple polynomial baseline using a Python script (see Supporting Information File 1). After baseline subtraction, the average power and standard deviation for the power were determined, as shown in Figure 3. Since transmission through quartz cuvettes and windows is not complete [48], we also corrected for the power loss.

The power of the LED was first determined without any quartz elements in the path between the collimator and the sensor. Then the LED power was determined with the insulated jacket for the cuvette holder and a cuvette containing solvent in place. The power loss at the sample was approximated to be half of the total power loss recorded for the system. Table S1 in Supporting Information File 1 gives an overview of the measured powers and averages. The loss was between 6–8%, with no marked dependence on the wavelength of the LED. The final power was taken to be the weighted mean of 3–4 measurements, using the reciprocal of the variance as the weights. The average error in the measured power is 10^−6^ W. These results grant a precision which is high enough to calculate the quantum yields, as our total error on the determined Φ is of the same order of magnitude as the ones earlier reported (vide infra) [22,24].

The current output of the LED driver is set by a manual dial, which adds another variable to the setup, inevitably causing some variation in the output power between different days. To avoid reproducibility errors, we measured the output current of the LED associated with a given power readout utilizing a multimeter connected to the LED driver. Nevertheless, we also checked the reproducibility of the measurements with different currents to test the stability of the setup (see Supporting Information File 1).

### Quantum yield determination

**Figure 4:**
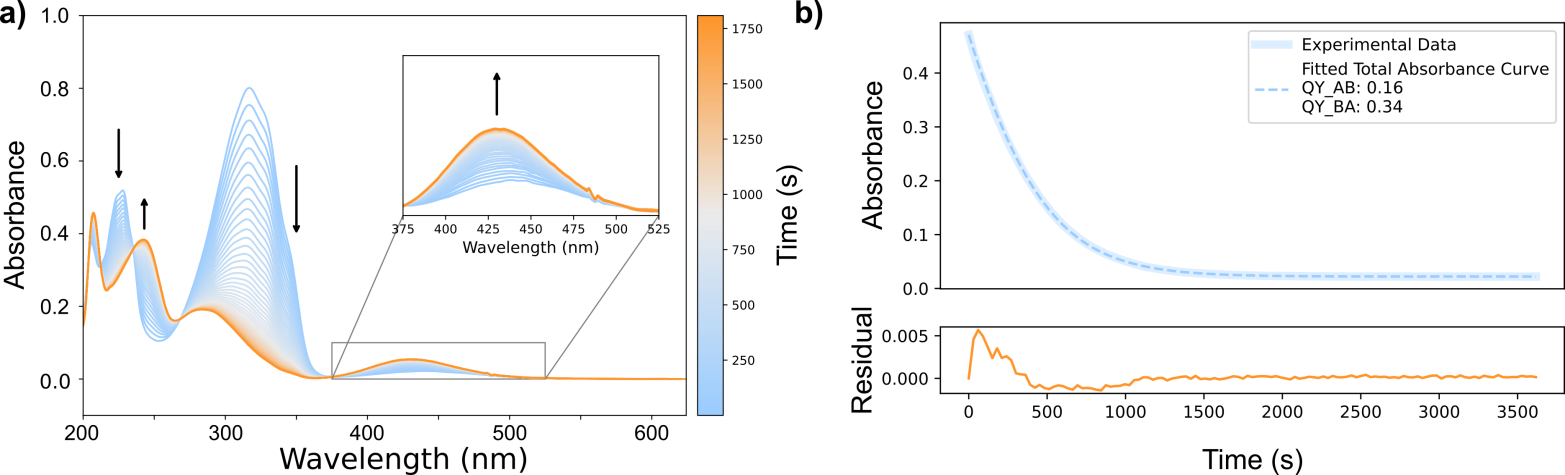
UV–vis absorption spectra of azobenzene upon irradiation at 340 nm (methanol solution, 20 °C). a) Evolution of spectra over time under irradiation and b) absorption at 340 nm over time, with fitted total absorbance curve obtained from Equation 5. The curve was obtained from entry 5 of Table S2 (Supporting Information File 1).

A concentrated solution of azobenzene (Sigma-Aldrich) in methanol (Supelco LiChrosolv) was prepared by dissolving an aliquot of crystalline azobenzene in 1.00 mL of solvent. Samples of 3.00 mL were prepared in a quartz cuvette (Hellma 117-100F QS) by diluting an aliquot of the concentrated solution in methanol, to obtain an absorbance of the π→π* band between 0.8 and 1.0. The samples of azobenzene were kept at 20 °C and stirred throughout the measurement (1200 rpm). The samples were irradiated for 1 h, while a UV–vis absorption spectrum was recorded every 30 s. The results for one such determination are shown in Figure 4A.

We utilized the powers obtained following the procedure described in the previous section to retrieve the quantum yields of isomerization, assuming strictly monochromatic light at first [24,49]. We chose the peak of the emission spectrum of the LED as the approximated monochromatic wavelength, since this value can substantially differ from the nominal wavelength of the LED, e.g., the 455 nm LED from ThorLabs has a peak in the emission spectrum at 445 nm. We furthermore determined the quantum yields again, taking the emission spectrum of the LED into account. The emission spectra were measured using our spectrometer by connecting the LEDs to the detector (Figure S1 in Supporting Information File 1). The following input parameters were used in both methods: the photon flux was calculated from the weighted mean of the power measured; molar absorptivities, ε_A_ and ε_B_, as well as the thermal rate constant, *k*_B→A_, were taken from the most recent study reporting these values [17]. The initial spectrum was confirmed to contain 100% *trans*-azobenzene by comparison with the known absorption spectrum of the pure switch [17].

To retrieve the quantum yield values for forward and backward isomerization, we solved rate equations Equations 5 and 6 (or Equation 6 and Equation 7, when the monochromatic excitation light approximation was not considered) numerically using two different python scripts (provided in Supporting Information File 1). The rate equations are ordinary differential equations (ODEs) and can thus be solved numerically using existing methods in the SciPy package [50]. An absorbance function was constructed, which takes the solution of the ODEs to model the absorbance over time. This absorbance function was then fitted to the measured absorbance at the wavelength of irradiation (or wavelengths, for a non-monochromatic excitation light). The parameters Φ_A→B_ and Φ_B→A_ were evaluated to minimize the residual sum of squares (RSS) between the measured and fitted absorbance values. This minimization is done by a Levenberg–Marquardt algorithm [51] as implemented in the lmfit package [52]. Using this package, the standard error of the fitted parameters is also obtained.

By evaluating the magnitude of the errors, we noticed that the largest source of uncertainty comes, not surprisingly, from the LED power measurement. This error is not included in the standard error of the fit, making the latter a poor reflection of the uncertainty in the retrieved quantum yields.

To overcome this limitation, we decided to evaluate the quantum yields using the powers corresponding to the lower and upper limits recorded with the thermopile. This procedure leads to two different values of quantum yields, Φ_min power_ and Φ_max power_, respectively, for both Φ_A→B_ and Φ_B→A_. In order to include the uncertainty of the fit (ΔΦ_fit)_, we corrected the thus obtained quantum yields using Equation 9 and Equation 10 to derive Φ_min_ and Φ_max_:


[9] $$\Phi_{\min}=\Phi_{\min\text{ power}}-\Delta \Phi_{\min\text{ power}}$$

[10] $$\Phi_{\max}=\Phi_{\max\text{ power}}+\Delta \Phi_{\max\text{ power}}$$

The total uncertainty on the quantum yield of a single measurement obtained by employing the weighted mean of the power (Φ_Α→Β_ in the following example), is:


[11] $$\Delta \Phi_{A\to B}=\max(\left|\Phi_{\text{A}\to \text{B}}-\Phi_{\min}\right|,\;\left|\Phi_{\text{A}\to \text{B}}-\Phi_{\max}\right|)$$

The values listed in Table 1 are the weighted means and sample standard deviations of multiple measurements, using the reciprocal of the variance of the quantum yields as the weights. For completeness, we report all the primary data that were utilized to compile Table 1 in Supporting Information File 1.

**Table 1:** Calculated quantum yields and related quantum yields from the literature.

Wavelength of irradiation (nm)		This work; single wavelength	This work; integrated emission	Ladányi et al. (2017) [24]	Gauglitz and Hubig (1985) [22]

340^a^/334^b^	*trans → cis*	0.160 ± 0.003	0.166 ± 0.003	0.155 ± 0.006	0.15 ± 0.02
	*cis → trans*	0.376 ± 0.070	0.384 ± 0.069	0.388 ± 0.053	0.30 ± 0.03
395^a^/405^b^	*trans → cis*	0.314 ± 0.050	0.264 ± 0.030	0.288 ± 0.007	0.20 ± 0.02
	*cis → trans*	0.578 ± 0.076	0.513 ± 0.040	0.452 ± 0.018	0.57 ± 0.03
436	*trans → cis*	–	–	0.315 ± 0.002	0.22 ± 0.03
	*cis → trans*	–	–	0.469 ± 0.003	0.63 ± 0.05
455	*trans → cis*	0.352 ± 0.003	0.354 ± 0.025		
	*cis → trans*	0.542 ± 0.034	0.569 ± 0.009		

^a^Irradiation used in this work; ^b^irradiation used in literature.

The measured quantum yields show good agreement with the values reported in the literature [22,24], especially for the 340 nm irradiation. It should be noted that an exact match of the quantum yields should not be expected, as the irradiation wavelengths are slightly different from the ones in the previous studies, because different excitation sources were used (non-monochromatic LED in our case, versus monochromatic light). In line with the findings of Ladányi et al., the precision of Φ*_trans_*_→_*_cis_* is generally better than the precision for the reverse photoreaction. This difference in precision could be attributed to the *trans→cis* reaction being more dominant, especially for irradiation in the π→π* band, where the absorption of *trans*-azobenzene is considerably higher than the *cis* (see Figure S4 in Supporting Information File 1). The use of Equation 7 to take the emission spectrum of the LED into account increased the quantum yield values by a few percent. The difference when using the integration is larger for Φ*_cis_*_→_*_trans_*. Since the emission of the LED has a larger bandwidth than the emission using a monochromator and filter, the integration over the bandwidth of the LED should be performed for the most accurate results. We would recommend measuring the emission spectra of the LEDs if possible. Although we did not find any significant changes between the quantum yields found using the measured emission spectra and the ones provided by the vendor, it is known that small variations in the LED spectral distribution can occur per lot [53].

## Conclusion

In this work, we report a simple setup for the determination of quantum yields using LEDs for irradiation and a thermopile detector for the direct determination of the power. The setup is built from commercially available optical elements and (together with the scripts provided in Supporting Information File 1) can be replicated at moderate financial costs. The inclusion of a temperature-controlled cuvette holder and the possibility for simultaneous UV–vis absorption spectroscopy makes this setup ideally suitable for a wide range of photoswitches, including those with fast thermal back isomerization. Our setup was tested by determining the quantum yields of azobenzene for different wavelengths of irradiation. The measured quantum yields showed good agreement with the most recently reported values [24], making this method and setup a viable alternative to actinometry.

## Supporting Information

File 1 Scripts description, configuration and supplementary data.

## Data Availability

The data generated and analyzed during this study is openly available in a figshare repository at https://doi.org/10.6084/m9.figshare.25818016.
