# Supplementary material for: A fiber-optic spectroscopic setup for isomerization quantum yield determination
Source: Beilstein J Org Chem. 2024 Jul 22;20:1684–92. doi: 10.3762/bjoc.20.150 (PMC11285046; doi:10.3762/bjoc.20.150)
Supplement: File 1 — Scripts description, configuration and supplementary data. [file Beilstein_J_Org_Chem-20-1684-s001.pdf]

BEILSTEIN JOURNAL OF ORGANIC CHEMISTRY

Supporting Information

for

A fiber-optic spectroscopic setup for isomerization quantum yield determination

Anouk Volker, Jorn D. Steen and Stefano Crespi

Beilstein J. Org. Chem. **2024,** *20,* 1684–1692. doi:10.3762/bjoc.20.150

Scripts description, configuration and supplementary data

License and Terms: This is a supporting information file under the terms of the Creative Commons Attribution License (https://creativecommons.org/licenses/by/4.0). Please note that the reuse, redistribution and reproduction in particular requires that the author(s) and source are credited and that individual graphics may be subject to special legal provisions.
The license is subject to the *Beilstein Journal of Organic Chemistry* terms and conditions: (https://www.beilstein-journals.org/bjoc/terms)

Table of contents

Scripts and configurations S1
Hardware required (main parts) S1
Software required S2
Overview of measurement steps S2
Configuration of irradiation setup S3
Configuration of AvaSoft S3
Connect the Arduino to the LED driver S3
Arduino control of LED driver S5
Run measurement with AutoHotkey script S6
Measure the current with a multimeter S7
Supplementary data S9
References S12

Scripts and configurations

All additional Python scripts, data examples, Arduino inputs and AutoHotkey setup files are stored in a figshare repository openly accessible at the following DOI: 10.6084/m9.figshare.25818016

Hardware required (main parts)

- Fiber-coupled UV–vis light source (with internal shutter)
- Fiber-coupled spectrometer (including interface capability with the light source)
- Enclosed cuvette holder (with four optical ports)
- Power meter (e.g., thermopile)
- Fiber-coupled LEDs
- LED driver with TTL trigger mode
- Arduino (e.g., UNO R3)

Software required

- AvaSoft[1]
- AutoHotkey[2]
- Python3
- Arduino IDE[3]

Overview of measurement steps

1. Open light source shutter (AvaSoft)
2. Acquire spectrum (AvaSoft)
3. Close light source shutter (AvaSoft)
4. Turn on LED (Arduino)
5. Wait for the user-defined interval time
6. Turn off LED (Arduino)

Repeat this for a user-defined number of cycles.

The measurement is controlled by an AutoHotkey[2] (v2.0) script that does two things:

- Acquire a spectrum by starting a *Single measurement* in AvaSoft,[1] which also controls the light source shutter (steps 1–3).
- Turn *on* or *off* the LED using a Python script to communicate with an Arduino UNO R3[4] that is connected to an LED driver[5] (steps 4–6).

Additionally, the script generates a log file that contains the timestamps of *acquisition*, *LED on*, and *LED off*.

Configuration of irradiation setup

- Configure AvaSoft to control the shutter of the light source. See Configuration of AvaSoft.
- Connect the Arduino to the LED driver with a BNC cable. See Connect the Arduino to the LED driver.
- Set up scripts for the control of the LED driver with the Arduino. See Arduino control of LED driver.

Configuration of AvaSoft

The light source (AvaLight-DH-S-BAL) is connected to the spectrometer via an interface cable (IC-DB26-RM), which allows AvaSoft to control the internal shutter of the light source. Set the shutter to be closed between measurements by checking the box *TTL Close Shutter Except when Measuring* in the *Digital IO* tab of the *Settings for spectrometer* window. With this configuration, starting a **single** measurement in AvaSoft sends a TTL signal to the light source that opens the shutter, which is followed by the acquisition of a single spectrum, and finally a TTL signal is sent to close the shutter again.

Connect the Arduino to the LED driver

For the connection of the Arduino with the LED driver, we stripped one end of a BNC cable to reveal two wires (live and ground) that we soldered to small metal pins that fit into the Arduino UNO R3 pins (Figure S1). The live wire is connected to PIN12 of the DIGITAL section, and the ground wire to GND of the POWER section on the Arduino circuit board.

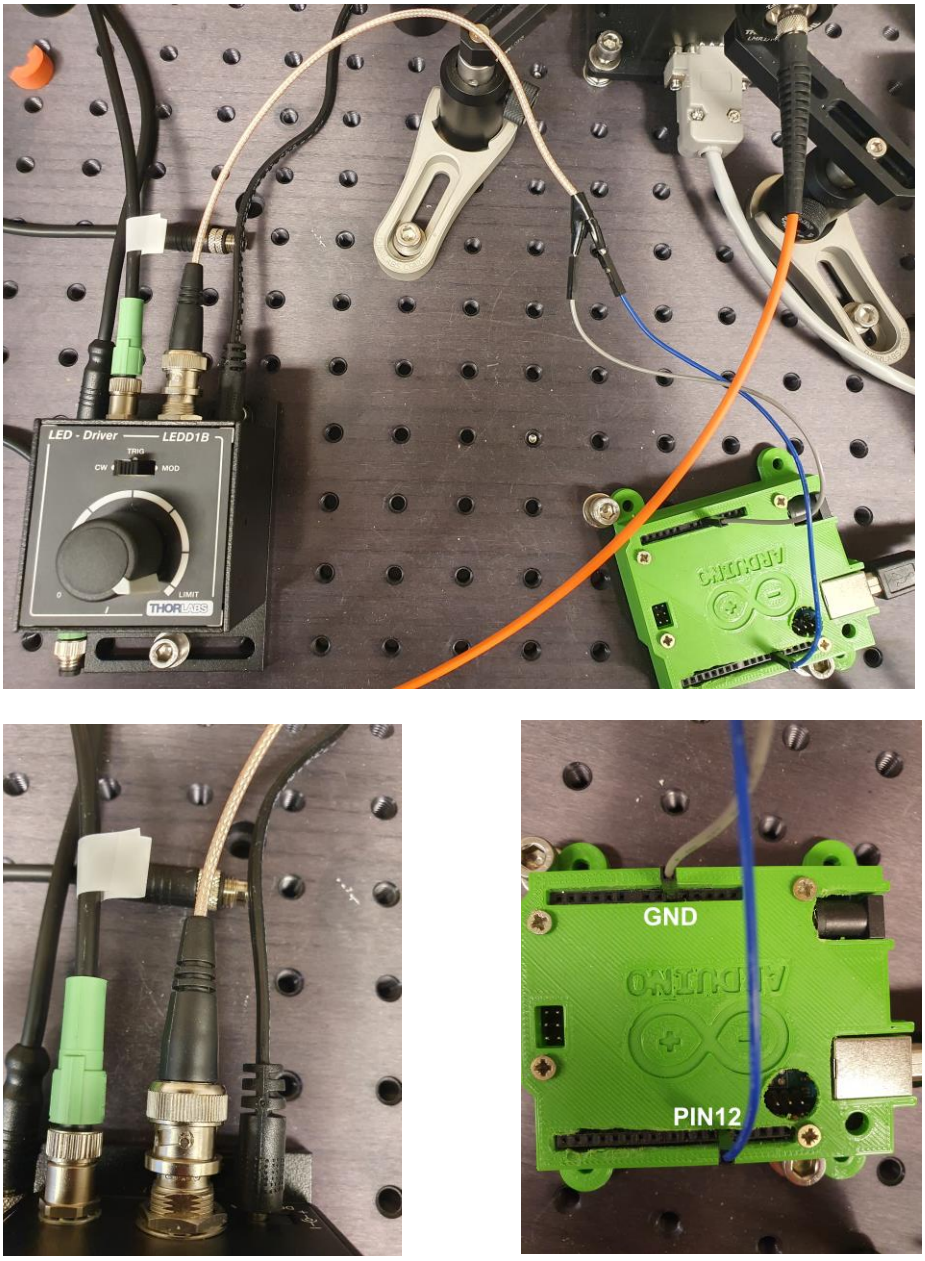

Figure S1: Pictures of the connection between the LED driver and the Arduino via a BNC cable that was stripped at one end in order to connect it to the pins GND and PIN12 of the Arduino.

Make sure the LED driver is set to TRIG mode (Figure S2). This setting means the LED driver will turn **off** upon receiving a 0 V TTL trigger, and **on** upon receiving a 5 V trigger from the Arduino.

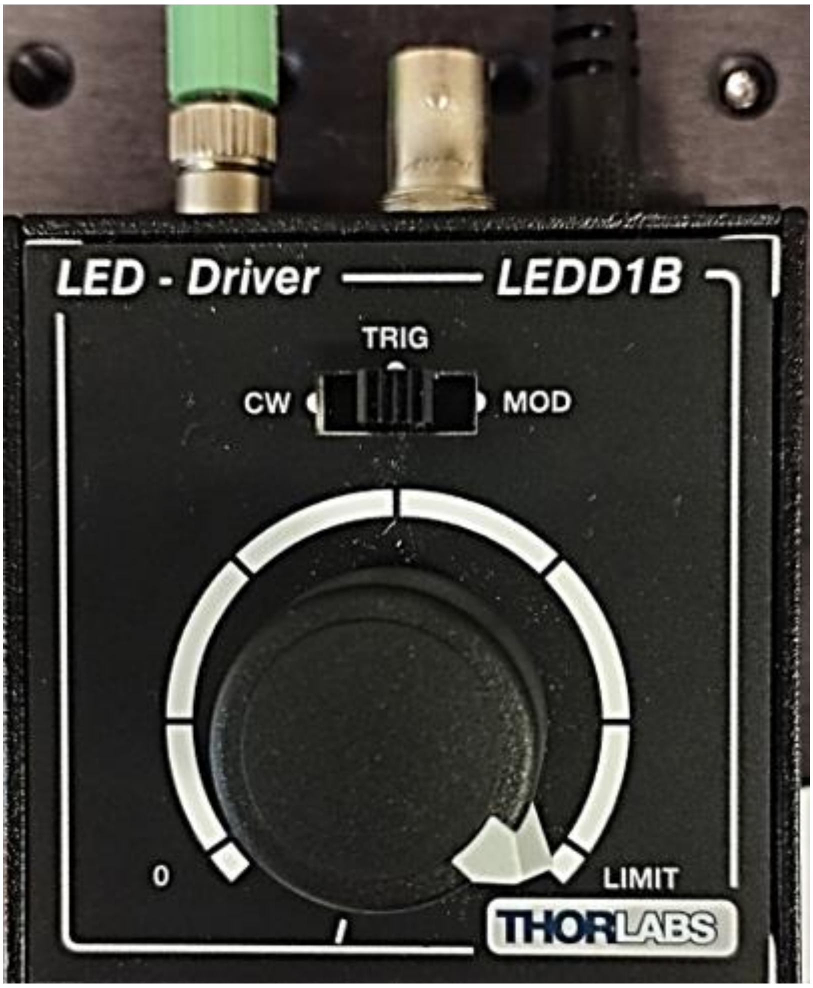

Figure S2: Picture of the LED driver in TRIG mode.

Arduino control of LED driver

1. Upload *SerialReadCOM.ino* to Arduino

This file needs to be uploaded to the Arduino before use (using the Arduino IDE[3]), in order for the Arduino to be able to receive commands from the computer by means of the Python script (see below). After it has been uploaded once, the script does not need to be uploaded again as Arduino will store the script in its internal memory.

2. Configure *ArduinoCommunication.py*
   a. Edit the script to use the correct COM port that is connected to the Arduino

b. Place the script in the same folder as that containing *Autoclick_IrrKin.ahk*

This Python script communicates with and sends commands to the Arduino *via* its COM port.

3. Modify and uncomment the line in *Autoclick_IrrKin.ahk* that changes the current working directory to the directory containing *ArduinoCommunication.py*, i.e. the same folder that contains the AutoHotkey script.

Run measurement with AutoHotkey script

1. Choose a fiber-optic LED with the desired emission wavelength
2. Connect fiber-optic cable used for irradiation to the LED
3. Connect LED power cable to the LED driver and set corresponding maximum current
    a. For measuring the current with a multi-meter: see Measure the current with a multimeter
    b. Make sure the LED driver is set to TRIG mode.
4. Open a Windows PowerShell window (it is needed by *Autoclick_IrrKin.ahk* to run *ArduinoCommunication.py*)
5. Make sure to set AvaSoft to collect single spectra (Start > Single measurement)
6. Set up the auto-save option in AvaSoft (File > Save to file)
7. Execute *Autoclick_IrrKin.ahk*
    a. Choose location and filename for log file
    b. Input the requested parameters: *interval* and *number of cycles*
8. Start the measurement (using the hotkey LCtrl+LAlt+.)

9. Check that the LED indeed turns on (and off) at the times that it is supposed to according to the input parameters.
10. If necessary, stop the measurement using the hotkey LCtrl+LAlt+,

Measure the current with a multimeter

We attached crocodile clips to the brown (Pin 1 Anode) and white (Pin 2 Cathode) wire ends of an LED connection cable (Thorlabs CAB-LEDD1) (Figure S3). These crocodile clips are used to clamp onto the probes of a multimeter in order to measure the current output of the LED driver.

N.B. be careful: high currents (up to 1.2 A).

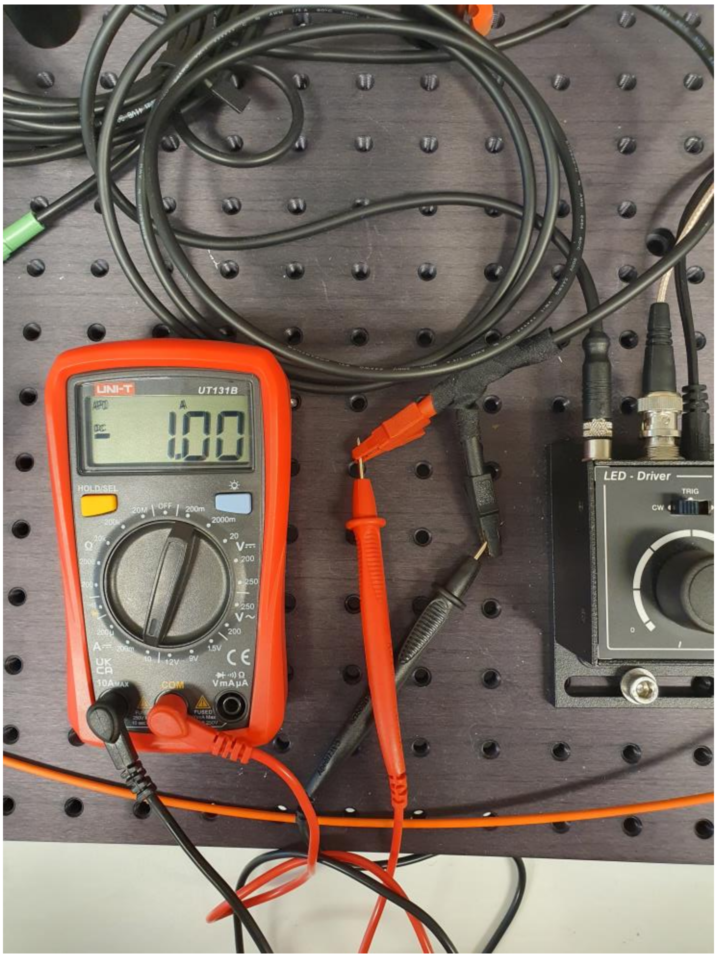

Figure S3: Picture of the LED connection cable modified with attached crocodile clips being used to measure the current output of the LED driver with a multimeter.

Supplementary data

Table S1: Power measurements for different LEDs. Differences in power for the 340 nm LED are due to different currents set for the LED driver, to test the reproducibility of the method.

Wavelength[a]	Repeat	Power without cover (mW)		Power with cover (mW)		Power loss[b] (mW)		Power loss[c] (%)	Power at sample (mW)	
		**value**	**σ**	**value**	**σ**	**value**	**σ**		**value**	**σ**
340	1	0.753	0.013	0.693	0.001	0.060	0.013	4	0.723	0.006
340	2	0.800	0.002	0.691	0.001	0.109	0.003	7	0.746	0.002
340	3	0.787	0.004	0.691	0.003	0.097	0.005	6	0.739	0.004
**weighted mean**									**0.743**	**0.002**
340	1	0.684	0.009	0.583	0.002	0.101	0.010	7	0.634	0.005
340	2	0.692	0.007	0.589	0.003	0.103	0.007	7	0.640	0.005
340	3	0.700	0.005	0.590	0.002	0.110	0.006	8	0.645	0.003
**weighted mean**									**0.641**	**0.002**
455	1	1.937	0.009	1.658	0.002	0.279	0.009	7	1.798	0.005
455	2	1.934	0.011	1.658	0.002	0.275	0.011	7	1.796	0.006
455	3	1.950	0.010	1.652	0.003	0.298	0.010	8	1.801	0.006
**weighted mean**									**1.798**	**0.003**
395	1	2.880	0.011	2.418	0.004	0.462	0.011	8	2.649	0.007
395	2	2.890	0.005	2.415	0.003	0.475	0.006	8	2.652	0.004
395	3	2.902	0.004	2.409	0.002	0.493	0.005	8	2.655	0.003
**weighted mean**									**2.654**	**0.002**

[a] nominal wavelength, [b] total power loss, [c] percentage power loss at sample

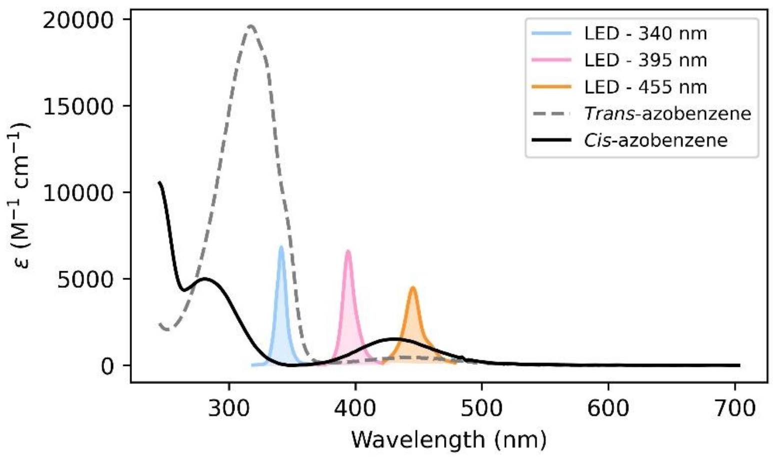

Figure S4: LED emission spectra of the ThorLabs LEDs used for quantum yield determination. Their emission is shown relative to the absorption bands of *cis*- and *trans*-azobenzene.

Table S2: Calculated quantum yields for single measurements and their obtained averages.

Wavelength[a]	Sample	Power (mW)			Φ Single wavelength		Φ Integrated emission	
		value	error		value	error[b]	value	error[b]
340	1	0.743	0.002	*trans → cis*	0.1607	0.0007	0.1660	0.0005
				cis → trans	0.331	0.004	0.338	0.002
	2	0.743	0.002	*trans → cis*	0.1556	0.0006	0.1609	0.0005
				cis → trans	0.389	0.004	0.392	0.002
	3	0.743	0.002	*trans → cis*	0.1619	0.0009	0.1669	0.0006
				cis → trans	0.518	0.008	0.519	0.003
	4	0.743	0.002	*trans → cis*	0.1600	0.0007	0.16509	0.0005
				cis → trans	0.391	0.005	0.387	0.002
	5	0.641	0.002	*trans → cis*	0.1638	0.0008	0.1692	0.0006
				cis → trans	0.340	0.002	0.344	0.002
	6	0.641	0.002	*trans → cis*	0.1645	0.0009	0.1702	0.0006
				cis → trans	0.449	0.004	0.448	0.002
**340**	**Weighted mean**			***trans → cis***	**0.160**	**0.003**	**0.166**	**0.003**
				cis → trans	**0.376**	**0.07**	**0.384**	**0.07**
455	1	1.798	0.003	*trans → cis*	0.348	0.009	0.386	0.003
				cis → trans	0.502	0.014	0.558	0.004
	2	1.798	0.003	*trans → cis*	0.353	0.007	0.335	0.002
				cis → trans	0.570	0.011	0.576	0.003
	3	1.798	0.003	*trans → cis*	0.353	0.006	0.361	0.002
				cis → trans	0.541	0.011	0.570	0.003
**455**	**Weighted mean**			***trans → cis***	**0.352**	**0.003**	**0.354**	**0.025**
				cis → trans	**0.542**	**0.034**	**0.569**	**0.009**
395	1	2.654	0.002	*trans → cis*	0.290	0.010	0.244	0.003
				cis → trans	0.508	0.019	0.473	0.006
	2	2.654	0.002	*trans → cis*	0.307	0.010	0.266	0.003
				cis → trans	0.645	0.024	0.543	0.007
	3	2.654	0.002	*trans → cis*	0.385	0.015	0.303	0.004
				cis → trans	0.635	0.027	0.540	0.008
**395**	**Weighted mean**			***trans → cis***	**0.314**	**0.050**	**0.264**	**0.030**
				cis → trans	**0.578**	**0.076**	**0.513**	**0.040**

[a] nominal wavelength, [b] error is calculated as the square root of the sample variance

References

1 AvaSoft Software, https://www.avantes.com/products/software/avasoft/, (accessed 13 May 2024).
2 AutoHotkey, https://www.autohotkey.com/, (accessed 13 May 2024).
3 Arduino Software, https://www.arduino.cc/en/software, (accessed 13 May 2024).
4 UNO R3 | Arduino Documentation, https://docs.arduino.cc/hardware/uno-rev3/, (accessed 13 May 2024).
5 Thorlabs - LEDD1B T-Cube LED Driver, 1200 mA Max Drive Current (Power Supply Not Included), https://www.thorlabs.com, (accessed 13 May 2024).
